# Insight Into the Origin and Evolution of the *Vibrio parahaemolyticus* Pandemic Strain

**DOI:** 10.3389/fmicb.2017.01397

**Published:** 2017-07-24

**Authors:** Romilio T. Espejo, Katherine García, Nicolas Plaza

**Affiliations:** ^1^Laboratory of Biotechnology, Institute of Nutrition and Food Technology, Universidad de Chile Santiago, Chile; ^2^Centro de Investigación Biomédica, Facultad de Ciencias de la Salud, Instituto de Ciencias Biomédicas, Universidad Autónoma de Chile Santiago, Chile

**Keywords:** genomic, phylogeny, diarrhea, ecology, horizontal gene transfer

## Abstract

A strain of *Vibrio parahaemolyticus* that emerged in 1995 caused the first known pandemic involving this species. This strain comprises clonal autochthonous ocean-dwelling bacteria whose evolution has occurred in the ocean environment. The low sequence diversity in this population enabled the discovery of information on its origin and evolution that has been hidden in bacterial clones that have evolved over a long period. Multilocus sequencing and microarray analysis, together with phylogenetic analysis, of pandemic and pre-pandemic isolates has suggested that the founder clone was an O3:K6 non-pathogenic strain that initially acquired a *toxRS*/new region and subsequently acquired at least seven novel genomic islands. Sequencing and comparison of whole genomes later confirmed these early observations, and it confirmed that most of the genetic changes occurred via gene conversion involving horizontally transmitted DNA. The highly clonal population rapidly diversified, especially in terms of antigenicity, and 27 serotypes have already been reported. Comparisons of the core genomes derived from the founder clone indicate that there are only a few hundred single-nucleotide variations between isolates. However, when the whole genome is considered (the core plus non-core genome and from any clonal frame), the amount of DNA with a different clonal frame can reach up to 4.2% and the number of single-nucleotide variations can reach several hundred thousand. Altogether, these and previous observations based on multilocus sequence typing, microarray analysis, and whole-genome sequencing indicate the large contribution made by DNA with different clonal genealogy to genome diversification. The evidence also indicates that horizontal gene transfer (HGT) caused the emergence of new pathogens. Furthermore, the extent of HGT seems to depend on the vicissitudes of the life of each bacterium, as exemplified by differences in thousands of base pairs acquired by HGT among almost identical genetic isolates.

## Introduction

Bacterial pathogens continuously cause problems because of the emergence of new pathogens and the evolution of existing pathogens. A pathogenic strain of *Vibrio parahaemolyticus* emerged in 1995 that caused the first pandemic in the history of this species. Ten years after its appearance in Southeast Asia, this pandemic strain caused one of the world’s worst diarrhea outbreaks in Chile, with more than 10,000 clinical cases. Clinical cases and the presence of the bacteria in seafood practically disappeared a few years later (García et al., 2013). Similar situations were observed in other world regions (DePaola et al., 2000, 2009; Tuyet et al., 2002; Chowdhury et al., 2004). The species *V. parahaemolyticus* includes autochthonous ocean-dwelling bacterial strains. Only a few strains, like the pandemic strain, can cause severe diarrhea when present in seafood (Letchumanan et al., 2014). Since the diarrhea is not transmitted person-to-person but by mollusks or other seafood contaminated with environmental bacteria, the emergence of new pathogens and also the disappearance of isolates is caused by evolution of this species in the ocean. Being pathogenic to humans in this case evolved by “coincidental” selection of traits beneficial for bacteria in the ocean that also conferred virulence in humans. The rise and fall of a strain in the ocean probably follows patterns that are common in evolution, defined by the ocean ecology.

The history of the pandemic strain began when a novel strain of *V. parahaemolyticus* with serovar O3:K6 was abundantly observed in Calcutta, India, in 1966 (Okuda et al., 1997). Analysis of 134 isolates obtained from January 1994 to August 1996 found that most isolates obtained after February 1996 had a particular pattern consisting of *tdh*+ (thermostable direct hemolysin gene), *trh*- (thermostable related hemolysin gene), urease+, and serovar O3:K6 (Okuda et al., 1997). The sharing of these properties and the similarity of the DNA of the isolates observed by an arbitrarily primed PCR method indicated that these isolates belonged to a unique clone, initially called serovar O3:K6. Further molecular analysis demonstrated other unique properties; among them, an associated bacteriophage with a unique open reading frame called *orf8* (Nasu et al., 2000), and a unique sequence of the *toxR* and *toxS* genes in the *toxRS* operon that encode transmembrane proteins involved in the regulation of virulence-associated genes. This specific sequence permitted the development of a PCR method that is exclusive for the pandemic strain, known as group-specific PCR (GS-PCR) (Kim et al., 1999). These genetic patterns and those obtained after restriction fragment length polymorphism–pulsed-field gel electrophoresis (RFLP-PFGE) of the DNA (Chowdhury et al., 2000) were employed to classify isolates from clinical sources in Taiwan, Laos, Japan, Thailand, Korea and the United States within this clonal group, showing its rapid dissemination worldwide (Matsumoto et al., 2000), and leading to the strain being designated as a pandemic strain of *V. parahaemolyticus*.

The whole genome of RIMD 2210633, isolated at the Kansai International Airport quarantine station in 1996, is now the reference genome of the pandemic strain (Makino et al., 2003). The sequence showed that the genome consists of two circular chromosomes of approximately 3.3 and 1.9 Mbp, chromosomes 1 and 2, respectively, with 4832 annotated proteins. Among the more interesting properties is the presence of an 81-kbp pathogenicity island present on chromosome 2, encoding a type three secretion system (T3SS) and two copies of the *tdh* gene, known to be associated only with pathogenic strains, a cytotoxic necrotizing factor, an exoenzyme T gene and five transposase genes. Also present is a large gene-capture system on chromosome 1, the super-integron (SI), found in various *Vibrios*.

## Origin of the Pandemic Strain

Bioinformatic and molecular analysis of the genome of RIMD 2210633 showed the presence of six additional genomic islands (Hurley et al., 2006), VPaI-1 to VPaI-7, with VPaI-7 being the island in chromosome 2 previously described by Makino et al. (2003). Analysis of 41 worldwide isolates of *V. parahaemolyticus* demonstrated that four of the islands (VPaI-1 and VPaI-4 to VPaI-6) were exclusive to the pandemic strain. This observation led to the conclusion that the pre-ancestral pandemic clone acquired these four islands, increasing both fitness in the ocean environment and ability to infect humans. Later, comparison with the genome of AQ3810 (Boyd et al., 2008), a *V. parahaemolyticus* O3:K6 isolate recovered in 1983 with extensive sequence homology to RIMD 2210633, showed that VPaI-1 to VPaI-6 are absent or partially missing in the pre-pandemic isolate. Also, missing in AQ3810 is a type VI secretion system (T6SS), identified in a range of Gram-negative pathogens including pathogenic *V. cholera*. A phylogenetic frame constructed from concatenated sequences of three housekeeping genes (*mdh*, *gyrB*, and *groEL*-1) from 42 isolates of *V. parahaemolyticus* from Asia, Europe and South America, encompassing 10 different serotypes, showed that VPaI-2 and VPaI-3 are predominantly present among pandemic strain isolates, similar to the previous observation for VPaI-1 and VPaI-4 to VPaI-6 (Hurley et al., 2006). From this work, the authors concluded that the most parsimonious scenario for the evolution of the pandemic strain clone was that a pre-1995 O3:K6 strain obtained regions VPaI-1 to VPaI-7, and a T6SS encoded within open reading frame (ORFs) VP1386–VP1420 by horizontal gene transmission. Blast analysis indicated that the possible origins of these regions would be quite diverse. A highly homologous VPaI-1-encoded protein was found in a 22-kbp island present in *V. cholera*. Other regions showed high similarities with those from other species: VPaI-3 to a region in *V. harveyi*, several ORFs of VPaI-2 to ORFs identified in *Vibrio* species, most of VPaI-5 to ORFs from *Shewanella woodyi*, several ORFs of VPaI-6 to a region in other species of the genus *Shewanella*, T3SS-2 to a T3SS in *V. cholera*, and VP1386 to VP1420, which encodes a T6SS, to a region in *V. harveyi* (Boyd et al., 2008).

Analysis of 174 global isolates by whole-genome cDNA microarray comparative genomic hybridization with amplicons from 4660 genes representing about 96% of the *V. parahaemolyticus* genes led to a similar proposal (Han et al., 2008). Phylogenetic analysis of the data assigned all the pandemic strain isolates (*trh^-^*, *tdh^+^*, and GS-PCR+) to the same highly conserved group called C3, while 12 pre-1996 O3:K6 strains (*trh^+^*, *tdh^-^* and GS-PCR-) were assigned to a different but also highly conserved group called C2. A minimum spanning tree based on the similarity matrix suggested that the pandemic strain emerged from O3:K6 clonal group C2. In fact, a possible phylogenetic intermediate group, *trh^-^*, *tdh^+^*, and GS-PCR+ (that acquired *toxRS*/new), differing from the clonal C2 by two loci, was identified. The authors confirmed the acquisition of genomic islands and proposed that the pandemic strain emerged from the old O3:K6 clone by the stepwise acquisition of genomic islands. A small group of O3:K6 strains (named as the intermediate-O3:K6 clade) served as the phylogenetic intermediate between new-O3:K6 and old-O3:K6.

Comparison of whole-genome sequences of AQ3810 and AQ4037 (another pre-pandemic O3:K6 *V. parahaemolyticus* obtained in 1985) with the reference genome of the pandemic strain RIMD 2210633, deepened our insight on the origin of the pandemic strain (Chen et al., 2011). Both, AQ3810 and AQ4037 are phylogenetically very close to RIMD 2210633, differing in approximately 28,000 single-nucleotide variations (SNV). However, they differ between themselves in 42,520 SNV (Chen et al., 2011, supplementary file 1), and VPaI*-7*, together with both copies of *tdh*, are absent in AQ4037. Interestingly, however, the structure of AQ3810 VPaI-7 is different to that found in the pandemic strain. These differences suggest that an ancestral strain possessing the O3:K6 serotype may have recruited a *tdh*-containing island.

**FIGURE 1:**
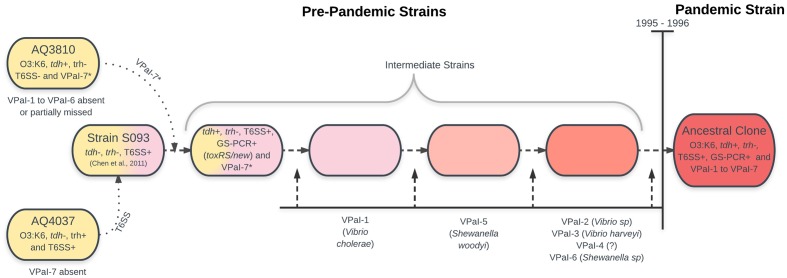
Schematic representation of a plausible origin of the pandemic *V. parahaemolyticus* clone founder bacteria. Ancestral bacteria on the left, intermediates from left to right. According to the text and references cited.

Taken together, the above observations suggest the origin of the pandemic strain is as shown in **Figure 1**.

## Evolution of the Pandemic Strain

Early analysis of a few genes (*tdh*, *trh*, *orf8*, and *toxRS*) and molecular techniques applied to isolates with serotype O3:K6 obtained after 1996 showed that they were undifferentiated, and the population was considered clonal (genes deriving from a same common ancestor) even though only a few genes were tested. Later analysis using a large number of isolates and techniques with higher resolution such as RFLP-PFGE (Chowdhury et al., 2000), direct genome restriction enzyme analysis (DGREA) by conventional gel electrophoresis (Fuenzalida et al., 2006), multilocus sequence typing (MLST) (Chowdhury et al., 2004; González-Escalona et al., 2008; Chao et al., 2011), multilocus variable-number tandem-repeat analysis (MLVA) (Kimura et al., 2008; García et al., 2012), microarray analysis (Izutsu et al., 2008) and finally whole-genome sequencing (Chen et al., 2011; Loyola et al., 2015, 2016), confirmed the clonal nature of the group. However, these techniques also showed the existence of variants that were considered evolutionary products of the initial clone, and showed numerous cases of genomic regions with numerous SNVs indicating origin from a different ancestor. Bacterial genomes evolve through two mechanisms: (1) mutations or occasional loss of ancestral genes, which preserve the founder clonal genealogy or frame; and (2) sporadic gains of new genes via horizontal gene transfer (HGT), which introduces DNA with a different genealogy or clonal frame (Milkman and Bridges, 1990).

Serotype changes were the earliest and most abundant polymorphisms observed in the pandemic clone. From 1996 to 2007, up to 22 pandemic serovariants were identified around the world (Nair et al., 2007). The latest report increased this number to 27 (Han et al., 2017), suggesting that the pandemic isolates could easily adopt new serotypes to survive in new environments. Whole-genome sequence analysis of three pandemic isolates, including one with a different serotype (O4:K68) (Chen et al., 2011), showed high similarity along the whole genome in this clonal complex except in the O- and K-antigen-encoding gene clusters, which contained 94% of the SNVs. Later, in ClonalframeML analysis of whole genomes, the O and K coding regions were identified as recombinant regions in three pandemic isolates with a serotype other than O3:K6 (Loyola et al., 2016). These and other less direct observations indicated that serotype conversion was due to a recombination or gene conversion event.

Besides the changes in serotype, analysis of specific genes showed that some genes once considered essential in the pandemic strain could be missing in some isolates. For example, the absence of phage f237 and hence *orf8* (Chowdhury et al., 2000; Bhuiyan et al., 2002) and VPaI-4 has been described (Chao et al., 2010). However, the pandemic strain is not only a receptor, genes of the pandemic strain can also be transferred to related bacteria, contributing to the evolution of bacteria in the local community. Four clinical isolates containing a VPaI-7 identical to the pandemic strain that prevails in the region, but differing in the rest of the genome, emerged in Chile in 2007 (Harth et al., 2009). Variable gene regions exclusive of the pandemic strain were observed in Peruvian isolates obtained after the arrival of the pandemic strain to Peru in 1977 (Gavilan et al., 2013).

MLST has been extensively applied for analysis of the pandemic strain population. Early MLST analysis with only four housekeeping genes (Chowdhury et al., 2000) already showed a clonal complex and also single-locus variants indicating early differentiation of the clone. A second MLST scheme including seven genes was established together with a centralized database^1^ (González-Escalona et al., 2008). This database contains, as of June 2017, sequences for 2525 isolates of *V. parahaemolyticus*. Another MLST analysis with 10 loci has also been employed (Yan et al., 2011). In general, these analyses together with eBurst (Feil et al., 2004) showed that most pandemic strain isolates cluster within a single clonal complex (CC3), with most showing the founder single sequence ST3 and multiple single and double locus variants (SLV and DSV). Recent MLST analysis of isolates from China showed 15 sequence types, revealing increasing genetic diversity among pandemic isolates, 10 of which fell within CC3 (Han et al., 2017). The isolates also showed frequent recombination among the genes or loci included in the MLST.

A similar view of this population was attained using microarray-based comparative genomic hybridization (M-GCH). Analysis of 4021 genes allowed clustering of 39 pandemic strains (defined as pandemic because they are *trh^-^*, *tdh^+^*, and GS-PCR+) in a single group called C3, which could be subdivided into five subgroups: SG1 to SG5, each containing one to 26 isolates (Han et al., 2008).

More recently, taking advantage of the high number of isolates with sequenced genomes, a core genome MLST (cgMLST) was designed, including 2254 core genes (Gonzalez-Escalona et al., 2017). Inclusion of O- and K-antigen coding genes allowed grouping of the strains in independent clusters according to their serotype. The high number of loci analyzed allowed observation of a high level of diversity within each cluster and was highly effective in separating strains from different outbreaks, in some cases distinguishing outbreaks caused by slightly different pandemic strains.

The resolving capacity of cgMLST seems only exceeded by whole-genome analysis (WGA). However, it is worth reviewing analysis of the highly variable number tandem repeats (VNTRs) before WGA. VNTRs consist of short sequences, known as repeat units or motifs, that are repeated in tandem and have been shown to vary in repeat copy number by the insertion or deletion of one or more repeat units. In the pandemic strain, mutation rates in this region are in the order of 10^-4^ mutations per generation (García et al., 2012), and thus multilocus variable analysis (MLVA) of the VNTRs in pandemic strain isolates allows differentiation of almost every independent isolate. Comparison of the number of repeats in eight VNTRs in 28 pandemic strain isolates produced 28 distinct VNTR patterns (Kimura et al., 2008). Analysis of 36 pandemic isolates belonging to the clonal complex isolated in Chile produced 26 patterns (Harth-Chu et al., 2009). Measuring the absolute number of repeats in each VNTR locus allowed the study of phylogeny and clustering of isolates according to their geographical origin (Ansede-Bermejo et al., 2010; García et al., 2012).

**FIGURE 2:**
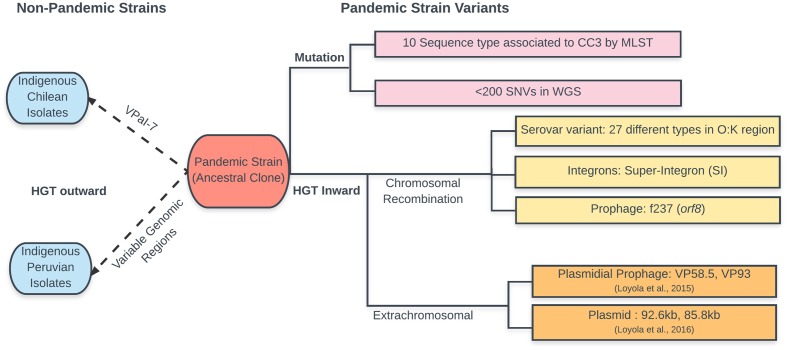
Schematic representation of the changes observed during evolution of the pandemic *V. parahaemolyticus* clone. According to the text and data in cited references. Changes in the ancestral clone are shown to the left separating mutations from HGT. Transmission of pandemic strain genes to other indigenous *V. parahaemolyticus* (in blue) is shown to the right of the ancestral clone.

Whole-genome comparisons of pandemic isolates have been published for three strains from three geographically distinct regions (Chen et al., 2011), eight from Chile (Loyola et al., 2015), and 31 worldwide isolates (Loyola et al., 2016). These studies have expanded our knowledge on the diversity and evolution of the pandemic strain. Initial comparison of three isolates (Chen et al., 2011) showed that major differences in the presence of pathogenicity islands and mobile elements are likely driving the evolution of pandemic *V. parahaemolyticus*. Accordingly, comparison of the core genomes in the eight Chilean isolates (i.e., genes shared in the eight isolates) showed small differences of only 13 to 164 SNVs. However, comparison of the genome length, including DNA not shared by all isolates, showed differences of 1366 to 217,729 bp, confirming that most differences corresponded to the presence of regions unique to only one or two isolates, probably acquired by HGT (Loyola et al., 2015). In some isolates, most of the non-shared DNA corresponded to extrachromosomal DNA. Genome innovation by the incorporation of unique DNA, attributable to HGT from related bacteria, varied greatly among these isolates. The large differences in the amount of non-shared DNA between highly similar isolates suggested that HGT appears to happen randomly within this group. This observation indicates the need for comparing the whole genome when studying evolution, incorporating exclusive DNA of each isolate and not only that shared by all isolates (core genome) which is used for building phylogenetic trees. Accordingly, a procedure called “reads accounting” was proposed when comparing genome sequences obtained by high-throughput sequencing (Loyola et al., 2016). This procedure aims to include in the comparison all the reads obtained after high-throughput sequencing of the bacterial DNA. It was used for genome comparison of 31 pandemic isolates obtained worldwide (Loyola et al., 2016). Further analysis of the clonal frames in the core genome of each isolate using ClonalFrameML (Didelot and Wilson, 2015) allowed inference of recombinant regions. When the whole genome is considered (core plus non-core genome), the relative amount of core genome passed clonally can be as low as 94.2%. However, when only the core genome is considered, the fraction retaining the founder clonal frame varied from 96.7 to 100%. The DNA with other clonal frames located in the chromosome, i.e., that which was horizontally transferred and recombined, was highly variable, ranging from 0.0 to 3.3%. The DNA not assigned to chromosomes, i.e., that obtained by HGT that did not recombine and remained as extrachromosmal DNA, varied from 0.0 to 4.2%. Taken together, these findings, and previous observations of MLST, microarray, and whole-genome sequencing, show the large contribution of DNA with different clonal genealogy to the diversification of the genomes and indicate that the emergence of new pathogens is primarily caused by HGT. The presence of isolates with exclusively pandemic clonal frame DNA and isolates with more than 100,000 bp of non-pandemic clonal frames suggest that extent of HGT depends on the vicissitudes of the life of each bacterium. In some isolates, these new DNA segments were in chromosomes, implying actual recombination, or gene conversion, while in other isolates, it was in extra-chromosomal elements. A schema of the evolution of the *V. parahaemolyticus* pandemic strain is shown in **Figure 2**.

Finally, it should be mentioned that a procedure for the comparison of the whole-genome sequence of *V. parahaemolyticus* is available from the National Center for Biotechnology Information^2^ (NCBI). This procedure provides cladograms and trees based on a pairwise Basic Local Alignment Search Tool (BLAST) comparison of chromosome sequences. However, there is no formal reference for this procedure and the intention is to provide a quick overview of the relationships, not a rigorous tree.

## Author Contributions

RE designed the minireview; RE and KG performed critical comparison of published literature and drafted the manuscript; KG and NP designed and drew the figures; RE, KG, and NP critically revised the manuscript.

## Conflict of Interest Statement

The authors declare that the research was conducted in the absence of any commercial or financial relationships that could be construed as a potential conflict of interest.

## Footnotes

1 http://pubmlst.org/vparahaemolyticus

2 https://www.ncbi.nlm.nih.gov/genome/691

## References

[B1] Ansede-Bermejo J. Gavilan R. G. Trinanes J. Espejo R. T. Martinez-Urtaza J. Ansede-Bermejo J. (2010). Origins and colonization history of pandemic *Vibrio parahaemolyticus* in South America. *Mol. Ecol.* 19 3924–3937. 10.1111/j.1365-294X.2010.04782.x 20735744

[B2] Bhuiyan N. A. Ansaruzzaman M. Kamruzzaman M. Alam K. Chowdhury N. R. Nishibuchi M. (2002). Prevalence of the pandemic genotype of *Vibrio parahaemolyticus* in Dhaka, Bangladesh, and significance of its distribution across different serotypes. *J. Clin. Microbiol.* 40 284–286. 10.1128/JCM.40.1.284-286.2002 11773134 PMC120132

[B3] Boyd E. F. Cohen A. L. V. Naughton L. M. Ussery D. W. Binnewies T. T. Stine O. C. (2008). Molecular analysis of the emergence of pandemic *Vibrio parahaemolyticus*. *BMC Microbiol.* 8:110 10.1186/1471-2180-8-110 PMC2491623 18590559

[B4] Chao G. Jiao X. Zhou X. Wang F. Yang Z. Huang J. (2010). Distribution of genes encoding four pathogenicity islands (VPaIs), T6SS, biofilm, and type I pilus in food and clinical strains of *Vibrio parahaemolyticus* in China. *Foodborne Pathog. Dis.* 7 649–658. 10.1089/fpd.2009.0441 20132020

[B5] Chao G. Wang F. Zhou X. Jiao X. Huang J. Pan Z. (2011). Origin of *Vibrio parahaemolyticus* O3:K6 pandemic clone. *Int. J. Food Microbiol.* 145 459–463. 10.1016/j.ijfoodmicro.2011.01.022 21316116

[B6] Chen Y. Stine O. C. Badger J. H. Gil A. I. Nair G. B. Nishibuchi M. (2011). Comparative genomic analysis of *Vibrio parahaemolyticus*: serotype conversion and virulence. *BMC Genomics* 12:294 10.1186/1471-2164-12-294 PMC3130711 21645368

[B7] Chowdhury N. R. Chakraborty S. Ramamurthy T. Nishibuchi M. Yamasaki S. Takeda Y. (2000). Molecular evidence of clonal *Vibrio parahaemolyticus* pandemic strains. *Emerg. Infect. Dis.* 6 631–636. 10.3201/eid0606.000612 11076722 PMC2640929

[B8] Chowdhury N. R. Stine O. C. Morris J. G. Nair G. B. (2004). Assessment of evolution of pandemic *Vibrio parahaemolyticus* by multilocus sequence typing. *J. Clin. Microbiol.* 42 1280–1282. 10.1128/JCM.42.3.1280-1282.2004 15004094 PMC356825

[B9] Depaola A. Jones J. L. Noe K. E. Byars R. H. Bowers J. C. (2009). Survey of postharvest-processed oysters in the United States for levels of *Vibrio vulnificus* and *Vibrio parahaemolyticus*. *J. Food Prot.* 72 2110–2113. 10.4315/0362-028X-72.10.2110 19833034

[B10] DePaola A. Kaysner C. A. Bowers J. Cook D. W. (2000). Environmental investigations of *Vibrio parahaemolyticus* in oysters after outbreaks in Washington, Texas, and New York (1997 and 1998). *Appl. Environ. Microbiol.* 66 4649–4654. 10.1128/AEM.66.11.4649-4654.2000 11055906 PMC92362

[B11] Didelot X. Wilson D. J. (2015). ClonalFrameML: efficient inference of recombination in whole bacterial genomes. *PLoS Comput. Biol.* 11:e1004041 10.1371/journal.pcbi.1004041 PMC4326465 25675341

[B12] Feil E. J. Li B. C. Aanensen D. M. Hanage W. P. Spratt B. G. (2004). eBURST: inferring patterns of evolutionary descent among clusters of related bacterial genotypes from multilocus sequence typing data. *J. Bacteriol.* 186 1518–1530. 10.1128/JB.186.5.1518-1530.2004 14973027 PMC344416

[B13] Fuenzalida L. Hernández C. Toro J. Rioseco M. L. Romero J. Espejo R. T. (2006). *Vibrio parahaemolyticus* in shellfish and clinical samples during two large epidemics of diarrhoea in southern Chile. *Environ. Microbiol.* 8 675–683. 10.1111/j.1462-2920.2005.00946.x 16584479

[B14] García K. Bastías R. Higuera G. Torres R. Mellado A. Uribe P. (2013). Rise and fall of pandemic *Vibrio parahaemolyticus* serotype O3:K6 in southern Chile. *Environ. Microbiol.* 15 527–534. 10.1111/j.1462-2920.2012.02883.x 23051148

[B15] García K. Gavilán R. G. Höfle M. G. Martínez-Urtaza J. Espejo R. T. (2012). Microevolution of pandemic *Vibrio parahaemolyticus* assessed by the number of repeat units in short sequence tandem repeat regions. *PLoS ONE* 7:e30823 10.1371/journal.pone.0030823 PMC3265528 22292049

[B16] Gavilan R. G. Zamudio M. L. Martinez-Urtaza J. (2013). Molecular epidemiology and genetic variation of pathogenic *Vibrio parahaemolyticus* in Peru. *PLoS Negl. Trop. Dis.* 7:e2210 10.1371/journal.pntd.0002210 PMC3656152 23696906

[B17] Gonzalez-Escalona N. Jolley K. A. Reed E. Martinez-Urtaza J. (2017). Defining a core genome multilocus sequence typing scheme for the global epidemiology of *Vibrio parahaemolyticus*. *J. Clin. Microbiol.* 55 1682–1697. 10.1128/JCM.00227-17 28330888 PMC5442524

[B18] González-Escalona N. Martinez-Urtaza J. Romero J. Espejo R. T. Jaykus L.-A. DePaola A. (2008). Determination of molecular phylogenetics of *Vibrio parahaemolyticus* strains by multilocus sequence typing. *J. Bacteriol.* 190 2831–2840. 10.1128/JB.01808-07 18281404 PMC2293261

[B19] Han D. Yu F. Tang H. Ren C. Wu C. Zhang P. (2017). Spreading of pandemic *Vibrio parahaemolyticus* O3:K6 and its serovariants: a re-analysis of strains isolated from multiple studies. *Front. Cell. Infect. Microbiol.* 7:188 10.3389/fcimb.2017.00188 PMC5435814 28573108

[B20] Han H. Wong H.-C. Kan B. Guo Z. Zeng X. Yin S. (2008). Genome plasticity of *Vibrio parahaemolyticus*: microevolution of the “pandemic group”. *BMC Genomics* 9:570 10.1186/1471-2164-9-570 PMC2612023 19038058

[B21] Harth E. Matsuda L. Hernández C. Rioseco M. L. Romero J. González-Escalona N. (2009). Epidemiology of *Vibrio parahaemolyticus* outbreaks, Southern Chile. *Emerg. Infect. Dis.* 15 163–168. 10.3201/eid1502.071269 19193258 PMC2657608

[B22] Harth-Chu E. Espejo R. T. Christen R. Guzmán C. A. Höfle M. G. (2009). Multiple-locus variable-number tandem-repeat analysis for clonal identification of *Vibrio parahaemolyticus* isolates by using capillary electrophoresis. *Appl. Environ. Microbiol.* 75 4079–4088. 10.1128/AEM.02729-08 19376898 PMC2698338

[B23] Hurley C. C. Quirke A. Reen F. J. Boyd E. F. (2006). Four genomic islands that mark post-1995 pandemic *Vibrio parahaemolyticus* isolates. *BMC Genomics* 7:104 10.1186/1471-2164-7-104 PMC1464126 16672049

[B24] Izutsu K. Kurokawa K. Tashiro K. Kuhara S. Hayashi T. Honda T. (2008). Comparative genomic analysis using microarray demonstrates a strong correlation between the presence of the 80-kilobase pathogenicity island and pathogenicity in Kanagawa phenomenon-positive *Vibrio parahaemolyticus* strains. *Infect. Immun.* 76 1016–1023. 10.1128/IAI.01535-07 18195030 PMC2258825

[B25] Kim Y. B. Okuda J. Matsumoto C. Takahashi N. Hashimoto S. Nishibuchi M. (1999). Identification of *Vibrio parahaemolyticus* strains at the species level by PCR targeted to the toxR gene. *J. Clin. Microbiol.* 37 1173–1177. 10074546 10.1128/jcm.37.4.1173-1177.1999 PMC88669

[B26] Kimura B. Sekine Y. Takahashi H. Tanaka Y. Obata H. Kai A. (2008). Multiple-locus variable-number of tandem-repeats analysis distinguishes *Vibrio parahaemolyticus* pandemic O3:K6 strains. *J. Microbiol. Methods* 72 313–320. 10.1016/j.mimet.2007.12.014 18258320

[B27] Letchumanan V. Chan K. G. Lee L. H. (2014). *Vibrio parahaemolyticus*: a review on the pathogenesis, prevalence, and advance molecular identification techniques. *Front. Microbiol.* 5:705 10.3389/fmicb.2014.00705 PMC4263241 25566219

[B28] Loyola D. E. Navarro C. Uribe P. García K. Mella C. Díaz D. (2015). Genome diversification within a clonal population of pandemic *Vibrio parahaemolyticus* seems to depend on the life circumstances of each individual bacteria. *BMC Genomics* 16:176 10.1186/s12864-015-1385-8 PMC4359782 25880192

[B29] Loyola D. E. Yañez C. Plaza N. García K. Espejo R. T. (2016). Genealogy of the genome components in the highly homogeneous pandemic *Vibrio parahaemolyticus* population. *J. Phylogenetics Evol. Biol.* 4:165 10.4172/2329-9002.1000165

[B30] Makino K. Oshima K. Kurokawa K. Yokoyama K. Uda T. Tagomori K. (2003). Genome sequence of *Vibrio parahaemolyticus*: a pathogenic mechanism distinct from that of *V. cholerae*. *Lancet* 361 743–749. 10.1016/S0140-6736(03)12659-1 12620739

[B31] Matsumoto C. Okuda J. Ishibashi M. Garg P. Rammamurthy T. Wong H. (2000). Pandemic spread of an O3: K6 Clone of *Vibrio parahaemolyticus* and emergence of related strains evidenced by arbitrarily primed PCR and *toxRS* sequence analyses. *J. Clin. Microbiol.* 2000 578–585. 10.1128/jcm.38.2.578-585.2000 PMC86152 10655349

[B32] Milkman R. Bridges M. M. (1990). Molecular evolution of the *Escherichia coli* chromosome. III. Clonal frames. *Genetics* 126 505–517. 1979037 10.1093/genetics/126.3.505 PMC1204208

[B33] Nair G. B. Ramamurthy T. Bhattacharya S. K. Dutta B. Takeda Y. Sack D. A. (2007). Global dissemination of *Vibrio parahaemolyticus* serotype O3:K6 and its serovariants. *Clin. Microbiol. Rev.* 20 39–48. 10.1128/CMR.00025-06 17223622 PMC1797631

[B34] Nasu H. Iida T. Sugahara T. Yamaichi Y. Park K. S. Yokoyama K. (2000). A filamentous phage associated with recent pandemic *Vibrio parahaemolyticus* O3:K6 strains. *J. Clin. Microbiol.* 38 2156–2161. 10834969 10.1128/jcm.38.6.2156-2161.2000 PMC86752

[B35] Okuda J. Ishibashi M. Hayakawa E. Nishino T. Takeda Y. Mukhopadhyay A. K. (1997). Emergence of a unique O3:K6 clone of *Vibrio parahaemolyticus* in Calcutta, India, and isolation of strains from the same clonal group from Southeast Asian travelers arriving in Japan. *J. Clin. Microbiol.* 35 3150–3155. 9399511 10.1128/jcm.35.12.3150-3155.1997 PMC230139

[B36] Tuyet D. T. Thiem V. D. Von Seidlein L. Chowdhury A. Park E. Canh D. G. (2002). Clinical, epidemiological, and socioeconomic analysis of an outbreak of *Vibrio parahaemolyticus* in Khanh Hoa Province. Vietnam. *J. Infect. Dis.* 186 1615–1620. 10.1086/345731 12447738

[B37] Yan Y. Cui Y. Han H. Xiao X. Wong H. C. Tan Y. (2011). Extended MLST-based population genetics and phylogeny of *Vibrio parahaemolyticus* with high levels of recombination. *Int. J. Food Microbiol.* 145 106–112. 10.1016/j.ijfoodmicro.2010.11.038 21176856

